# Direct Contact – Sorptive Tape Extraction coupled with Gas Chromatography – Mass Spectrometry to reveal volatile topographical dynamics of lima bean (*Phaseolus lunatus* L.) upon herbivory by *Spodoptera littoralis* Boisd.

**DOI:** 10.1186/s12870-015-0487-4

**Published:** 2015-04-12

**Authors:** Lorenzo Boggia, Barbara Sgorbini, Cinzia M Bertea, Cecilia Cagliero, Carlo Bicchi, Massimo E Maffei, Patrizia Rubiolo

**Affiliations:** Department of Drug Science and Technology, University of Turin, Via P. Giuria 9, 10125 Turin, Italy; Plant Physiology Unit, Department Life Sciences and Systems Biology, University of Turin, Via Quarello 15/A, 10135 Turin, Italy

**Keywords:** Direct Contact-Sorptive Tape Extraction (DC-STE), Gas Chromatography coupled with Mass Spectrometry (GC-MS), Herbivory-induced plant volatile (HIPV), *Phaseolus lunatus* L., *Spodoptera littoralis* Boisd., Plant-insect interactions, Herbivory, Green leaf volatiles (GLVs), Monoterpenoids, Sesquiterpenoids

## Abstract

**Background:**

The dynamics of plant volatile (PV) emission, and the relationship between damaged area and biosynthesis of bioactive molecules in plant-insect interactions, remain open questions. Direct Contact-Sorptive Tape Extraction (DC-STE) is a sorption sampling technique employing non adhesive polydimethylsiloxane tapes, which are placed in direct contact with a biologically-active surface. DC-STE coupled to Gas Chromatography – Mass Spectrometry (GC-MS) is a non-destructive, high concentration-capacity sampling technique able to detect and allow identification of PVs involved in plant responses to biotic and abiotic stresses. Here we investigated the leaf topographical dynamics of herbivory-induced PV (HIPV) produced by *Phaseolus lunatus* L. (lima bean) in response to herbivory by larvae of the Mediterranean climbing cutworm (*Spodoptera littoralis* Boisd.) and mechanical wounding by DC-STE-GC-MS.

**Results:**

Time-course experiments on herbivory wounding caused by larvae (HW), mechanical damage by a pattern wheel (MD), and MD combined with the larvae oral secretions (OS) showed that green leaf volatiles (GLVs) [(*E*)-2-hexenal, (*Z*)-3-hexen-1-ol, 1-octen-3-ol, (*Z*)-3-hexenyl acetate, (*Z*)-3-hexenyl butyrate] were associated with both MD and HW, whereas monoterpenoids [(*E*)-β-ocimene], sesquiterpenoids [(*E*)-nerolidol] and homoterpenes (DMNT and TMTT) were specifically associated with HW. Up-regulation of genes coding for HIPV-related enzymes (Farnesyl Pyrophosphate Synthase, Lipoxygenase, Ocimene Synthase and Terpene Synthase 2) was consistent with HIPV results. GLVs and sesquiterpenoids were produced locally and found to influence their own gene expression in distant tissues, whereas (*E*)-β-ocimene, TMTT, and DMNT gene expression was limited to wounded areas.

**Conclusions:**

DC-STE-GC-MS was found to be a reliable method for the topographical evaluation of plant responses to biotic and abiotic stresses, by revealing the differential distribution of different classes of HIPVs. The main advantages of this technique include: a) *in vivo* sampling; b) reproducible sampling; c) ease of execution; d) simultaneous assays of different leaf portions, and e) preservation of plant material for further “omic” studies. DC-STE-GC-MS is also a low-impact innovative method for *in situ* PV detection that finds potential applications in sustainable crop management.

**Electronic supplementary material:**

The online version of this article (doi:10.1186/s12870-015-0487-4) contains supplementary material, which is available to authorized users.

## Background

In the past ten years, the study of the interaction between larvae of the Mediterranean climbing cutworm (*Spodoptera littoralis* Boisd.) and leaves of the lima bean (*Phaseolus lunatus* L.) has provided evidence of both early and late events, and has been used as a model system to decipher plant-insect interactions [1-5]. Upon herbivory by *S. littoralis*, the lima bean responds, as do many other plants, with a cascade of events that lead to the activation of defense mechanisms. These mechanisms include the perception of molecular patterns or effectors of defense [6,7], mitogen-activated protein kinase (MAPK) activation, and protein phosphorylation [8,9], production of ethylene and jasmonates [10], expression of late defense response genes [11], and emission of herbivory-induced plant volatiles (HIPVs) [12,13].

Even if robotic mechanical wounding can simulate plant response similar to HIPV [4], the simple mechanical damage (MD) is not fully satisfactory to induce the same responses if not supported by the application of insect’s oral secretions (OS) [14]. Despite the presence of several elicitors in *S. littoralis* OS (e.g., fatty acid conjugates) [7,15], it is not clear whether these factors originate from the salivary glands or other feeding-related organs, such as the ventral eversible gland [14,16]. However, the plant volatile (PV) blends emitted in response to herbivores differ markedly with different feeding modes [17-20].

In plant defensive strategies, the release of PVs plays multiple roles: direct deterrents against herbivores [21,22], attraction of natural enemies of the attacking herbivores [23-26], damage and disease long-distance signaling [27-30], and pathogen resistance priming [29-32]. Since volatiles are produced from several biosynthetic pathways, their qualitative and quantitative composition is the result of the concerted action of different pathways, triggered by multiple factors. To date, studies of the emission of PVs in response to herbivory have been limited to single organs or to the whole plant, either by destructive methods or by head-space analysis [33,34], and only one study analyzed PV gradients within a single leaf [35].

**Figure 1:**
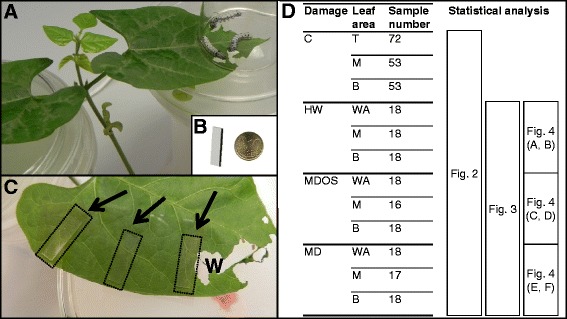
DC-STE sampling visualization. **A**, *Spodoptera littoralis* larvae feeding on *Phaseolus lunatus*. **B**, tape dimension. **C**, DC-STE tapes placed on the adaxial lamina of the wounded leaf. Arrows indicate the squared translucent tapes; W, wounded zone. **D**, Experimental and data analysis scheme; for every treatment, the number of analyzed samples is reported.

Direct Contact-Sorptive Tape Extraction (DC-STE) is a fast and easy-to-use sampling technique, developed to study the effect of cosmetic treatment on sebum composition, through *in vivo* sampling at the human skin surface [36,37]. The technique employs a thin flexible non-adhesive polydimethylsiloxane (PDMS) tape, which is placed directly in contact with a (biological) surface for a fixed time (Figure 1). Bicchi et al. [38] showed that this technique can also be applied to plants to monitor PVs, in both surface-static headspace and direct-contact (DC) modes. In DC-STE, volatiles produced at the biological surface are concentrated in the apolar PDMS layer by sorption (a sampling approach based on the partition of a compound between the sample and the bulk of a polymeric retaining phase) in amounts depending on the compound polarity and volatility. While in headspace sampling (e.g. static and dynamic headspace, high concentration-capacity solid phase microextraction) sorption is applied to the plant surrounding air space [33], DC-STE interacts directly with leaf surfaces. In DC-STE, plant-air interaction equilibrium is eliminated thus limiting the number of phases involved with sampling to two (plant and PDMS) instead of three (plant, air and PDMS). In this study, a glass coverslip was placed just above the DC-STE tape in order to exclude PDMS – air interaction.

Compound recovery from PDMS is achieved either by thermal desorption and on-line transferred to the injector of a Gas Chromatography–Mass Spectrometry (GC-MS) system, or by liquid extraction with polar solvents. DC-STE can be used successfully for both qualitative and quantitative analyses [37] making DC-STE coupled with GC-MS an efficient approach to characterize the profile and dynamics of PV production in response to both biotic and abiotic stresses.

In this study, the use of DC-STE combined with GC-MS was applied *in vivo* for the first time to evaluate the dynamics of HIPV release, upon abiotic (MD) and biotic (herbivory wounding, HW) stresses, by using the model system *S. littoralis*/*P. lunatus*. Furthermore, MD was used in combination with *S. littoralis* OS (MDOS). Here we show that HIPVs are differentially produced in different parts of the wounded leaf, depending on the biotic or abiotic stress applied. The analytical method was compared to the expression of genes involved in HIPV biosynthesis, which showed the same HIPV topographical pattern.

## Results

In response to herbivory, plants produce PVs, which can serve as direct deterrents [21] or to attract the herbivore’s predators and parasitoids [23-26,39]. The dynamics of HIPV emission, and the relationship between damaged area and biosynthesis of bioactive molecules, remain open questions. An innovative *in vivo* strategy was here used to identify compounds actively related to plant-insect interactions, employing a non-destructive high concentration-capacity sampling technique to capture volatiles from lima bean leaves after abiotic and biotic wounding.

### DC-STE-GC-MS analysis discriminates herbivory from mechanical wounding

To analyze the topographical distribution of HIPVs, leaves from plants grown in a growth chamber treated with HW, MD and MDOS as well as control intact leaves were sampled with PDMS rectangular tapes (4 × 15 × 0.2 mm) placed in direct contact with leaves at specific distances from the damaged areas (0 cm, 1.5 cm, 3 cm) for different sampling times (2, 6, 24 h). Adaxial and abaxial leaf laminae were sampled in three different leaf portions: a) close to the damaged area (referred as the wounding zone, 0 cm); b) in the central portion (referred as the middle zone, 1.5 cm); and c) in the basal portion of the leaf (referred as the basal zone, 3 cm) (Figure 1). Preliminary trials showed no significant differences in PV results between adaxial and abaxial epidermises (data not shown). Analysis of camphor variation supports the repeatability of the method, accounting for 18.3% as relative standard deviation throughout the whole dataset.

Several PVs were identified by GC-MS analyses including green-leaf volatiles (GLVs, including aldehydes, alcohols and acetates), alkyl aldehydes, homoterpenes, mono- and sesquiterpenoids (Additional file 1). Because of the large number of samples (337), several Principal Component Analyses (PCA) were carried out; the best results were those obtained with logarithmic scaling as data pre-treatment [40].

**Figure 2:**
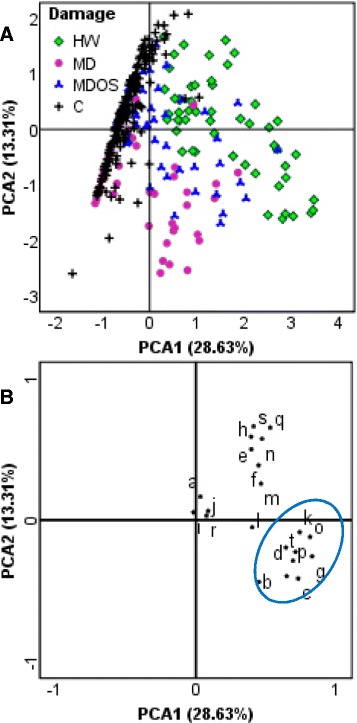
PCA representing the whole set of data. 337 samples are here plotted in PCA by using all compounds as variables. **A**, Control unwounded leaves (C) are well-separated from damaged leaves. HW and MD show a clear separation. MDOS produced intermediate patterns between HW and MD. **B**, Loading plot highlights the discriminant variables (blue circle). Compound legend: a, n-hexanal; b, (*E*)-2-hexenal; c, (*Z*)-3-hexen-1-ol; d, 1-octen-3-ol; e, 6-methyl-5-hepten-2-one; f, octanal; g, (*Z*)-3-hexenyl acetate; h, *p*-cymene; i, limonene; j, 2-ethyl hexanol; k, (*E*)-β-ocimene; l, 1-octanol; m, linalool; n, nonanal; o, DMNT; p, (*Z*)-3-hexenyl butyrate; q, decanal; r, tridecane; s, geranyl acetone; t, (*E*)-nerolidol; u, TMTT.

Figure 2A reports the PCA (42% of explained variance) on the total dataset of samples, discriminating undamaged (controls) from damaged leaves. The damaged sample distribution in Figure 2A showed that HW and MD seemed divided into two different subsets, while application of OS to MD leaves produced intermediate results between them.

**Figure 3:**
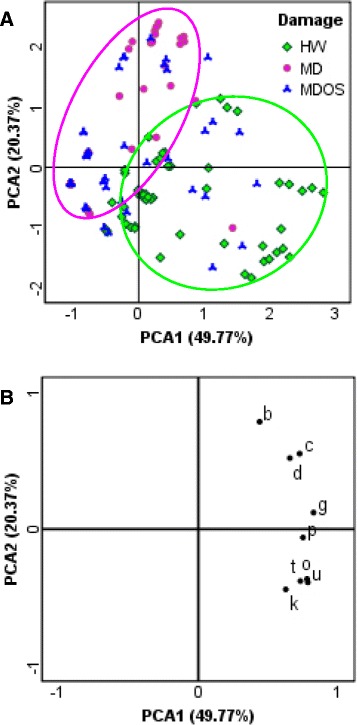
Damage sample dataset PCA. This analysis was done on the damaged samples with the variables selected in the first PCA. **A**, There is a clear distinction between HW (green squares) and MD (magenta circles) samples. Application of OS to MD (MDOS) produced scattered results. **B**, Loading plot. Compound legend: b, (*E*)-2-hexenal; c, (*Z*)-3-hexen-1-ol; d, 1-octen-3-ol; g, (*Z*)-3-hexenyl acetate; k, (*E*)-β-ocimene; o, DMNT; p, (*Z*)-3-hexenyl butyrate; t, (*E*)-nerolidol; u, TMTT.

The resulting damage-related discriminant compounds included GLVs [(*E*)-2-hexenal, (*Z*)-3-hexen-1-ol, (*Z*)-3-hexenyl acetate, (*Z*)-3-hexenyl butyrate], a linoleic acid breakdown product (1-octen-3-ol), a monoterpene [(*E*)*-*β-ocimene], two homoterpenes [4,8-dimethyl-1,3,7-nonatriene (DMNT) and 4,8,12-trimethyl-1,3,7,11-tridecatetraene (TMTT)] and a sesquiterpenoid [(*E*)-nerolidol] (Figure 2B). These HIPVs were therefore used as variables for the subsequent PCA to explore the internal differences in the damaged leaf dataset. A better discrimination (about 71% of total variance explained) was obtained between HW and MD treatments, whereas MDOS samples showed a scattered pattern (Figure 3).

### DC-STE-GC-MS determines and quantifies the topography of leaf HIPV production

The ability to discriminate between MD and HW highlights the potential of DC-STE-GC-MS as a reliable technique for *in vivo* HIPV monitoring. This ability was used to study the dynamics of volatile production as a function of topography in lima bean leaf responses to HW, MD and MDOS.

**Figure 4:**
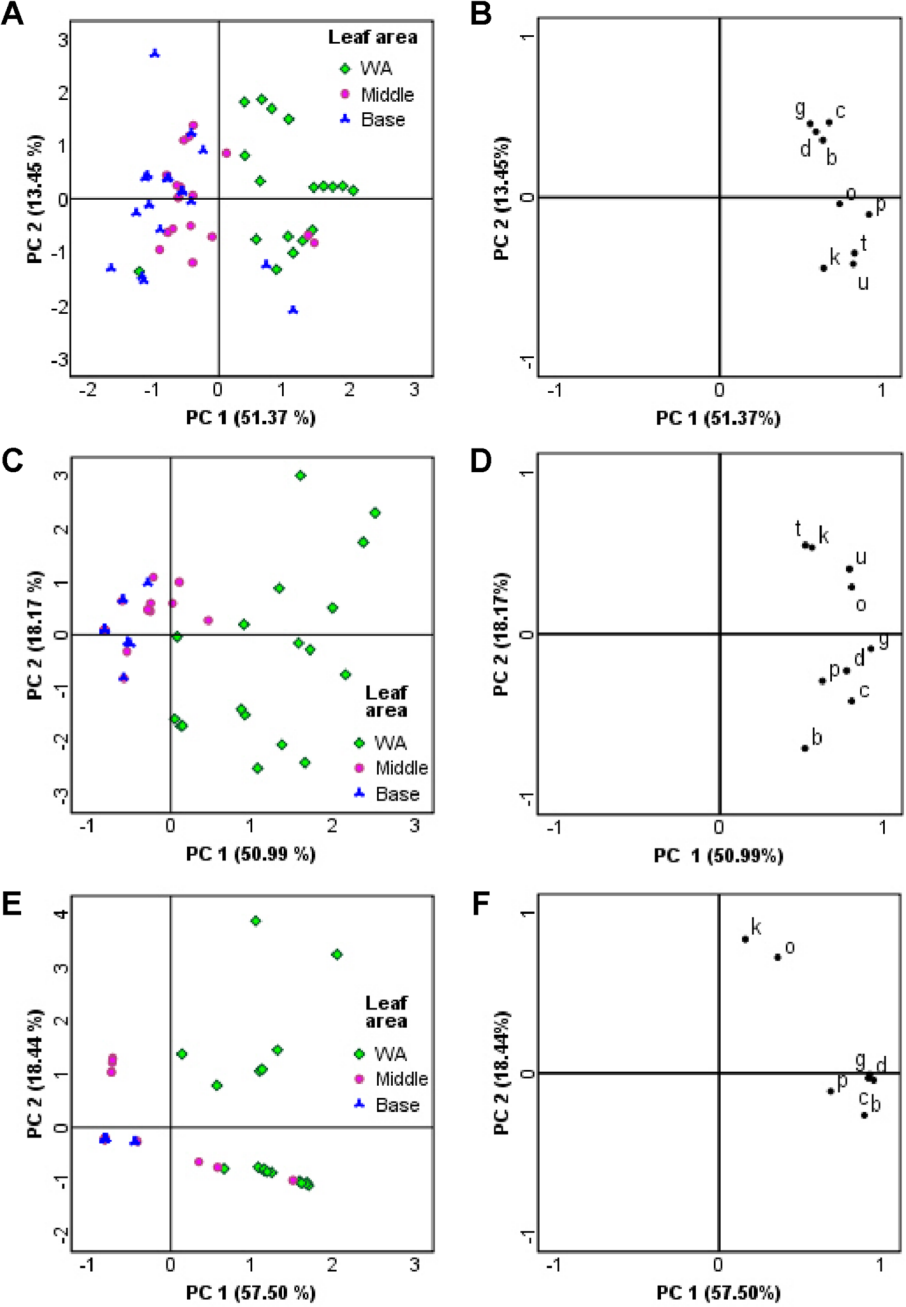
HIPV topography. HIPV topography is clearly shown in PCA score plots: wounded areas (WA) are in all cases clearly separated from other leaf portions. PCAs were carried out using: b, (*E*)-2-hexenal; c, (*Z*)-3-hexen-1-ol; d, 1-octen-3-ol; g, (*Z*)-3-hexenyl acetate; k, (*E*)-β-ocimene; o, DMNT; p, (*Z*)-3-hexenyl butyrate; t, (*E*)-nerolidol; u, TMTT. **A**, Score plot for HW leaves (54 samples) shows the distinction between WA samples (green squares) and other leaf portions. **B**, HW loading plot suggests that GLVs and terpenoids have the same localization in HIPV topographical distribution. **C**, MDOS leaves (52 samples) show a distribution similar to HW leaves (A). **D**, MDOS loading plot. **E**, MD score plot (53 samples) shows the same topographical distribution. **F**, MD loading plot shows a different distribution between GLVs and the terpenoid groups. The position of (k) and (o) and the absence of (t) and (u) suggest the non-significant role of terpenoids in MD reaction, unlike the HW and MDOS loading plots (B, D).

To visualize HIPV distribution, the damaged leaf dataset was divided into three different matrices, depending on the type of damage, each including a smaller but still considerable number of samples (HW: 54 samples; MD: 53 samples; MDOS: 52 samples). PCA data processing was performed by using the discriminating variables identified above (GLVs, homoterpenes, mono- and sesquiterpenoids) with the aim of establishing a relationship between sampling time and leaf portion. A distinctive distribution of volatiles as a function of the damaged area was found for both HW and MDOS (Figure 4: A and C). Compared to controls, HW treated leaves showed a significantly higher production of the GLVs (*E*)-2-hexenal, (*Z*)-3-hexen-1-ol, (*Z*)-3-hexenyl acetate and of 1-octen-3-ol close to the HW damaged leaf portion (Figure 4B). (*Z*)-3-Hexenyl butyrate, DMNT, TMTT, (*E*)-β-ocimene and (*E*)-nerolidol were produced in the same area, close to the HW zone, but also in distant leaf portions (Figure 4B). A similar pattern was found when MD plants were treated with OS (Figure 4: C and D). In MD treated leaves, there was a clear distinction between the wounded area and the rest of the leaf (Figure 4E). However, only GLVs and 1-octen-3-ol were produced in wounded areas, while (*E*)-β-ocimene and DMNT were not discriminant for the different leaf portions (Figure 4F).

The observation of the temporal differences showed that in all treatments GLVs were always produced early, whereas production of terpenoids and homoterpenes occurred later. In particular, PCAs highlighted some interesting differences in the temporal patterns between HW and other damages, with MDOS again showing intermediate values (Additional file 2).

A quantitative evaluation of the main damage-related compounds were carried out by combining in-tape camphor standardization with an external calibration by Gas Chromatography - Selected Ion Monitoring - Mass Spectrometry (GC-SIM-MS) for all types of damage, reaching a good linearity for every quantified HIPV (for quantitation parameters see Additional file 3).

In general, GLVs were the most abundant compounds in the damaged area (Table 1). (*Z*)-3-hexen-1-ol, (*E*)-2-hexenal, and 1-octen-3-ol reach rates of up to 100 ng/cm^2^. (*E*)-β-ocimene and (*E*)-nerolidol were generally produced in smaller amounts far from the wounded zone; however, they were found to exceed 100 ng/cm^2^ in the damage area. The homoterpenes, DMNT and TMTT, were mostly found in low quantities in HW-damaged leaves (Table 1).

**Table 1:** ***Phaseolus lunatus*** **HIPV quantitation results**

		**(** ***E*** **)-2-hexenal**	**(** ***Z*** **)-3-hexen-1-ol**	**1-octen-3-ol**	**(** ***Z*** **)-3-hexenyl acetate**	**(** ***E*** **)-β-ocimene**	**DMNT**	**(** ***Z*** **)-3-hexenyl butyrate**	**(** ***E*** **)-nerolidol**	**TMTT**
**HW**	**WA**	**185.4**	**341.1**	**152.8**	**65.8**	**274.3**	**62.2**	670.0	**237.7**	**63.6**
		**(45.6)**	**(67.5)**	**(51.8)**	**(11.3)**	**(53.2)**	**(8.0)**	(539.7)	**(82.2)**	**(23.6)**
	**M**	nd	38.8	42.6	29.2	111.7	24.7	40.6	40.6	12.6
			(9.9)	(12.6)	(10.7)	(38.5)	(5.8)	(31.0)	(30.1)	(6.3)
	**B**	nd	58.3	28.3	12.2	90.6	24.7	25.5	31.9	6.3
			(31.5)	(10.0)	(2.3)	(49.1)	(12.2)	(22.1)	(24.0)	(4.6)
**MDOS**	**WA**	**354.6**	**366.1**	**195.5**	**9.0**	**68**	**16.3**	7.6	63.8	**16.2**
		**(98.7)**	**(95.6)**	**(39.1)**	**(1.2)**	**(23.6)**	**(3.7)**	(5.8)	(40.5)	**(4.5)**
	**M**	9.8	18.5	20.6	nd	**45.6**	3.8	nd	nd	nd
		(9.8)	(12.8)	(8.4)		**(19.3)**	(1.7)			
	**B**	7.7	nd	8.2	nd	3.3	0.7	nd	nd	nd
		(7.7)		(4.7)		(1.8)	(0.7)			
**MD**	**WA**	**652.3**	**1436.4**	**435.1**	**12.5**	**277.6**	2.5	**11.6**	7.9	1.8
		**(157.7)**	**(220.0)**	**(101.4)**	**(1.7)**	**(120.2)**	(1.3)	**(5.5)**	(7.9)	(1.8)
	**M**	49.8	71.4	51.3	1.4	26.9	nd	nd	nd	nd
		(29.1)	(47.2)	(35.7)	(1.0)	(13.1)				
	**B**	nd	4.1	2.4	nd	nd	nd	nd	nd	nd
			(4.1)	(2.4)						
**Contr**	**T**	3.0	nd	1.6	0.2	nd	0.6	nd	nd	nd
		(3.0)		(1.1)	(0.2)		(0.4)			
	**M**	4.0	3.6	6.9	nd	nd	nd	nd	15.2	nd
		(4.0)	(2.5)	(2.7)					(12.2)	
	**B**	13.9	4.0	2.5	0.4	0.3	nd	nd	nd	nd
		(9.9)	(2.8)	(1.4)	(0.3)	(0.3)				

Quantitative analysis of HIPVs produced by *Phaseolus lunatus* in different stress conditions, and topographical distribution of HIPVs. Results are expressed as ng/cm^2^ (SEM). Reported results were submitted to ANOVA. Numbers in bold indicate statistical significance at the Tukey HSD test of the indicated leaf area (p < 0.05) in those cases in which ANOVA (treatments-control) was significant (p < 0.05). WA, wounded area; M, middle portion of the leaf; B, basal portion of the leaf; T, unwounded tip of the leaf; HW, herbivore wounding; MD, mechanical damage; MDOS, mechanical damage plus application of *Spodoptera littoralis* oral secretions; Contr, control undamaged leaves; nd, not detectable.

### Topographical gene expression analysis and DC-STE-GC-MS HIPV mapping

Because of the non-destructive DC-STE method of PV sampling, the different leaf sampled portions producing HIPVs could be used for gene expression analyses. Farnesyl Pyrophosphate Synthase (*FPS*) [41], *P. lunatus* Ocimene Synthase (*PlOS*) [10] and *P. lunatus* Terpene Synthase 2 (*PlTPS2*) [42] gene expressions were analyzed and compared to the results obtained by DC-STE for the related compounds . In addition, Lipoxygenase (*LOX*) [41] gene expression was analyzed, to assess any similarity with the observed high formation of GLVs.

**Figure 5:**
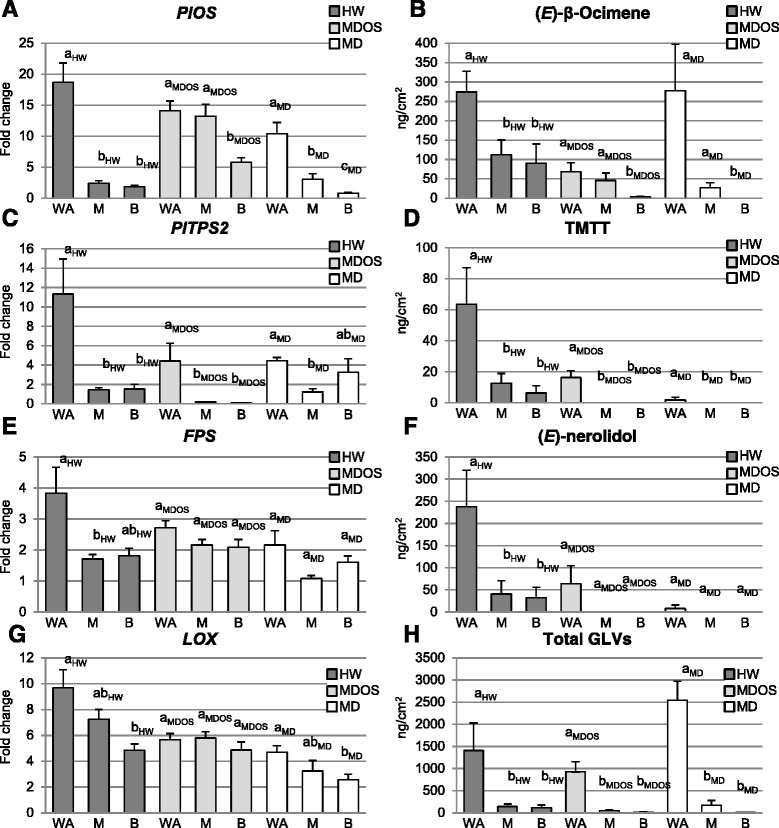
HIPV– gene expression comparison. Letters refer to Kruskal-Wallis tests conducted separately for each damage dataset (Bonferroni correction was applied, only p < 0.017 were accepted as significant). Comparison of HIPV quantitation results, and percentage change versus significance groups, point to a correlation between HIPVs and their biosynthesis distribution, and thus support the DC-STE-GC-MS results. WA, wounded area; M, middle; B, base. **A**, **C**, **E**, **G**: *PlOS*, *PlTPS2*, *FPS*, *LOX* quantitative real time-PCR (qPCR) calculated fold changes. **B**, **D**, **F**, **H**: (*E*)-β-ocimene, TMTT, (*E*)-nerolidol, total GLV quantitation results (ng/cm^2^).

Significantly higher expression of *PlOS* (Figure 5A) was in all cases coherent with the measured amount of the related compound (*E*)-β-ocimene (Figure 5B), with fold change values > 10 in the wounded zones of leaves treated by HW, MD or MDOS. Production of the homoterpene TMTT was associated with the gene expression pattern of *PlTPS2* only for HW and MDOS treatments, whereas regulation of the gene was not comparable to the amount of the homoterpene upon MD treatment (Figure 5: C and D). Upregulation of *FPS* gene expression (Figure 5E) was consistent with (*E*)-nerolidol amount in HW and MDOS treatments (Figure 5F). Finally, the total GLV - production (Figure 5H) was in all cases higher in wounded zones, and consistent with *LOX* upregulation, in particular when referred to HW (Figure 5G). These results are fully supported by the Kruskal-Wallis significance test (with Bonferroni adjustment, p < 0.017), as shown in Figure 5.

## Discussion

One of the most challenging tasks in multitrophic interaction studies is the adoption of advanced analytical platforms that enable different analyses to be run simultaneously using different “omic” methodologies. DC-STE-GC-MS enabled to characterize the qualitative and quantitative topographical profile of leaf volatile emission upon herbivory, while evaluating at the same time the gene expression of the same sampled tissues.

In general, the HIPVs detected upon biotic and abiotic stresses in this study agree with those associated with biological damaging events [9,13,22,43] and with indirect plant defense [20,25,30,44].

The present results highlight the key role of the damaged area in HIPV production [35], with GLVs associated with both mechanical damage and herbivory, and monoterpenoids, sesquiterpenoids, and homoterpenes specifically associated with herbivory. In particular, MD treatment appears to be sufficient to induce higher amount of GLVs, including (*E*)-2-hexenal, (*Z*)-3-hexen-1-ol, 1-octen-3-ol, (*Z*)-3-hexenyl acetate and (*Z*)-3-hexenyl butyrate [20,25,32,45-48]. The DC-STE-GC-MS technique enabled GLVs to be determined qualitatively and to be quantified for further comparisons. Furthermore, the analysis revealed that some GLVs [(*E*)-2-hexenal, (*Z*)-3-hexen-1-ol and 1-octen-3-ol] are more intensively produced during MD than they are during other stresses. Conversely, (*Z*)-3-hexenyl acetate and (*Z*)-3-hexenyl butyrate are produced in higher amount in HW leaves, supporting their herbivory-induced production pattern [23,25,26,49]. GLVs are synthesized via the *LOX* pathway from C_18_ polyunsaturated fatty acids [50], which are cleaved to C_12_ and C_6_ compounds by hydroperoxide lyases (HPL) [28]. Most plants have several isoforms of *LOX* [51], and a specific *LOX* that is essential to GLV formation has been identified in a few plant species [52]. In the present study, upregulation of *LOX* expression was evenly distributed throughout the leaf, although GLVs were mostly found in the wounded area. This discrepancy between gene expression and GLV production may be due, on the one hand, to the wide variety of roles played by *LOX* [53], and, on the other hand, to the effect of the GLVs on leaf tissues [30]. For instance, (*Z*)-3-hexenal in the vapor phase was taken up by *Arabidopsis* and converted into its alcohol and acetate in the cells. This scenario was further confirmed by the fact that the isotope ratios of alcohol and acetate were almost identical to that of (*Z*)-3-hexenal when ^13^C-labeled (*Z*)-3-hexenal of a given isotope ratio was used for the exposure [54]. GLVs produced in the wounded zone may therefore influence expression of genes in unwounded tissues of the same leaf because of GLV diffusion. In line with what Heil and Land have been recently reported [30], DC-STE sampling also highlights that GLVs play a central role in the so-called plant damage associated molecular pattern (DAMP). Indeed they seem to be essential to trigger gene expression required to prepare an adequate damage reaction in the surrounding tissues and organs. The MD related high GLV production could be explained with their well-known anti-microbial activity [32,55]. This is a resistance trait that is required during pathogen infection, which could occur after wounding [30], even without the herbivore interaction. Among monoterpenes, (*E*)-β-ocimene, a well-known damage-related HIPV [5,10,44] is a significant example of HIPV distribution. Its amount is limited to the damaged area in HW, while in MDOS (*E*)-β-ocimene also occurs distant from the wounded tissues. This different distribution agrees with the pattern of *PlOS* expression, demonstrating to produce almost exclusively (*E*)-β-ocimene when activated [56]; production is mainly located in the wounding area [45]. Transgenic *Arabidopsis*, transformed with the *PlOS* promoter *GUS* fusion constructs, shows that the activity is restricted to the wounded sites [10]. Lepidopteran caterpillars continuously remove leaf tissue after every bite, even if in a time longer than that one needed for the induction [57]. Conversely, application of OS to MD enables the elicitor to remain on the leaf longer, at least throughout the sampling time. This might explain why, in MDOS treated leaves, *PlOS* upregulation was observed in leaf areas distant from the damage.

Homoterpenes and sesquiterpenoids, such as DMNT, TMTT and (*E*)-nerolidol, are often associated with damage-related emission [5,22,26,58]; they have been studied as indirect defense mediators [25,39]. DMNT distribution is comparable to that of TMTT, and shows a general distribution from the damage zone throughout the leaf. However, their amount is higher in the wounded zone after both HW and MDOS treatment. The TPS enzymes have been found to be involved in DMNT and TMTT precursor production [42,58,59] and their products have been related to herbivory events [10,35,58]. The *PlTPS2* gene analyzed here showed a distribution comparable to that of homoterpene amount, in particular in leaves undergoing HW and MDOS.

Production of (*E*)-nerolidol is limited to the wounded zone, in particular in HW and MDOS damage, while MD does not seem to induce it. The lower amount of this compound in MDOS compared to HW is of interest because it shows the inability of OS alone to trigger the same HW-related leaf emission. Expression of *FPS* was found to be upregulated not only in damaged areas but also in leaf tissues distant from the wounding zone. *FPS* plays a key role in HIPV emission since its product, farnesyl pyrophosphate, is a basic precursor for sesquiterpenoid biosynthesis [13,60]. FPS is considered an important HW-related enzyme [43] and its inducibility by HIPVs has also been discussed and confirmed [61,62]. *FPS* upregulation was marked in HW leaves, underlining the relationship between herbivory and *FPS* activation [43].

## Conclusions

The use of DC-STE-GC-MS provides a clearer picture of DAMP distribution in lima bean, by showing differential release of HIPV classes after different kinds of wounding. DAMPs, which are essential for airborne damage-signals, were found to be mainly related to disrupted tissues. The results confirm the role of HIPVs as DAMP signals and show their role as signals able to quickly spread in the surrounding environment of wounded areas. Upon herbivory a fast *V*_*m*_ depolarization is known to affect the whole damaged leaf, whereas calcium, potassium, ROS and NO responses are limited to the wounded zones. DC-STE-GC-MS results show that GLVs are released almost immediately and their emission is topographically in concomitance with early events such as *V*_*m*_ depolarization and calcium signaling, as previous data suggested [32,63-69].

The DC-STE-GC-MS results are in agreement with the present body of knowledge of plant damage recognition and reaction, and provide a better understanding of the dynamics of plant responses to damage. The main advantages of this technique compared to classical PV sampling methods are: a) *in vivo* sampling; b) ease of execution; c) simultaneous assays of different leaf portions, and d) preservation of plant material for further omic studies.

## Methods

### Plant and animal material

Feeding experiments were carried out using the lima bean (*Phaseolus lunatus* L. cv Ferry Morse var. Jackson Wonder Bush). Individual plants were grown from seed in plastic pots with quartz sand at 23°C and 60% humidity, using daylight fluorescent tubes at approximately 270 μE m^−2^ s^−1^ with a photophase of 16 h. Experiments were conducted with 12- to 16-day-old seedlings showing two fully-developed primary leaves, which were found to be the most responsive [1].

*Spodoptera littoralis* Boisd. (Lepidoptera, Noctuidae) larvae were kindly provided by R. Reist from Syngenta Crop. Protection Münchwilen AG, Switzerland, and were fed on an artificial diet comprising 125 g bean flour, 2.25 g ascorbic acid, 2.25 g ethyl 4-hydroxybenzoate, 750 μL formaldehyde, 300 mL distilled water and 20 g agar, previously solubilized in 300 mL of distilled water. The ingredients (Sigma-Aldrich, St. Louis, MO, USA) were mixed with a blender and stored at 4°C for not more than one week. With the exception of plant volatile (PV) collection (see below), plants were exposed for 2 h to third instar larvae reared from egg clutches in Petri dishes (9 cm diameter) in a growth chamber with 16 h photoperiod at 25°C and 60-70% humidity. The amount of herbivore damage was limited to 30% of leaf surface, as detected by ImageJ image analysis [4]. Feeding experiments were always performed between 1 and 3 p.m.

### Collection of oral secretions

In order to evaluate the effect of *S. littoralis* oral secretions (OS), 5-day-old larvae were allowed to feed on lima bean leaves for 24 h. Regurgitation was caused by gently squeezing the larva with a forceps behind the head. OS was collected in glass capillaries connected to an evacuated sterile vial (peristaltic pump).

### PV sampling setup

Biotic stress was caused by *S. littoralis* (HW); whereas abiotic stress was performed by mechanically damaging leaf tissues with a pattern wheel (MD). Furthermore abiotic and biotic stresses were connected by combining MD with *S. littoralis* oral secretions (MDOS). A large number of samples were analyzed (337) and multivariate methods were used to define discriminant variables (i.e., HIPVs) and to plot chemical and molecular topographical maps of leaf areas producing HIPVs in response to biotic and abiotic stress. In particular, the experiments were carried out in nine sampling steps, each representing a specific combination of type of damage (HW, MD, and MDOS) and sampling duration (2, 6, 24 h). For each sampling step, three biological replicates were analyzed, with 12 tapes for each. A control using two tapes was also sampled. HW was caused by *S. littoralis* caterpillars; the damaged area for each plant was as near as possible equal. MD was done by piercing the leaves manually with a pattern wheel. The damaged leaf area and the duration of time of the damaging mechanism were kept constant. The damage process in MDOS was similar to that in MD, with the addition on the wounded area of 10 μL of a solution 1:1 of *S. littoralis* OS and 5 mM MES (2-(N-morpholino)-ethane-sulphonic acid) buffer (pH 6.0). The OS quantity was assessed after several trials (from 0.5 to 10 μL) and was found the most appropriate to obtain reproducible experiments [43].

At the end of the sampling time, the tapes were removed and stored at −20°C. Leaves were cut into 3 parts (wounded area, middle, base) and stored at −80°C for further analyses.

### Direct Contact–Sorptive Tape Extraction of PVs

Polydimethylsiloxane (PDMS) tapes (4 × 15 × 0.2 mm, *ca.* 33 mg) were placed on different areas of the adaxial and abaxial leaf lamina of *S. littoralis-*attacked and of control leaves. A glass coverslip was placed just above the DC-STE tape in order to exclude PDMS – air interaction. The quantitation of the collected PVs was obtained by an external standard at known concentration levels, being difficult to calculate an analyte recovery rate with DC-STE applied to *in vivo* plant matrices (unlike it was done in [37] with standards). Sampling was carried out in triplicate in the positions on the leaf shown in Figure 1, for the times reported above (2, 6, 24 h). Camphor (Sigma-Aldrich, Milan, Italy) was used as internal standard (I.S.) and was sorbed onto the tapes as proposed by Wang et al. [70] for Solid Phase Micro Extraction. Preliminary analysis with tapes with and without camphor I.S. were carried out to verify any possible interference of camphor with lima bean PV production (Additional file 4). After sampling, the PDMS tapes were placed in thermal desorption tubes, stored in sealed vials, and submitted to automatic thermal desorption (see below). Sorption tapes were provided by the Research Institute for Chromatography (Kortrijk-Belgium).

### GC-MS analysis

PDMS tape thermal desorption was carried out with a Thermal Desorption Unit (TDU) from Gerstel (Mülheima/d Ruhr, Germany). Analyses were driven automatically by an MPS-2 multipurpose sampler installed on an Agilent 7890 GC unit coupled to an Agilent 5975C MSD (Agilent, Little Falls, DE, USA). The TDU thermal desorption program was: from 30°C to 250°C (5 min) at 60°C/min in splitless flow mode, and transfer line at 300°C. A Gerstel CIS-4 PTV injector was used to cryofocus compounds thermally desorbed from the PDMS tapes, and inject them into the injector GC port. The PTV was cooled to −40°C using liquid CO_2_; injection temperature: from −40°C to 250°C (5 min) at 12°C/s. The inlet was operating in the splitless mode. Helium was used as carrier gas at a flow rate of 1 mL/min. Column: HP5MS (30 m × 0.25 mm i.d. × 0.25 μm; Agilent Technologies). Temperature program: from −30°C (1 min) to 50°C at 50°C/min, then to 165°C at 3°C/min, then to 250°C (5 min) at 25°C/min. MS operated in EI mode at 70 eV with a mass range from 35 to 350 amu in full scan mode.

Quantitative Gas Chromatography – Selected Ion Monitoring – Mass Spectrometry analysis (GC-SIM-MS): appropriate amounts of 2-hexenal, 3-hexenol, 1-octen-3-ol, 3-hexenyl acetate, (*Z*)-3-hexenyl butyrate, 1-octen-3-ol, (*E*)*-*β-ocimene, 4,8-dimethyl-1,3,7-nonatriene and 4,8,12-trimethyl-1,3,7,11-tridecatetraene (Sigma-Aldrich, Milan, Italy) were diluted with cyclohexane (Sigma-Aldrich, Milan, Italy) to obtain nine different concentrations in the range 1 to 1000 μg/mL for each component. Calibration curves were constructed by analyzing the resulting standard solutions three times, by GC-MS in SIM mode, under the conditions reported above.

### GC-MS data processing

Data were processed with Agilent MSD ChemStation *ver*. D.03.00.611 (Agilent Technologies). Components were identified by comparing their linear retention indices (*I*^*T*^s) (calculated *versus* a C_9_-C_25_ hydrocarbon mixture) and their mass spectra to those of authentic samples, or by comparison with those present in commercially-available mass spectrum libraries (Wiley, Adams).

### RNA extraction from lima bean leaves after HW, MD and MDOS

After each experiment, leaves were collected and immediately frozen in liquid nitrogen. Samples from time-course experiments were pooled so as to have a single pool of replicates for each stress condition (HW, MDOS, MD, undamaged leaves). Fifty mg of frozen leaf material were ground in liquid nitrogen with mortar and pestle. Total RNA was isolated using the Agilent Plant RNA Isolation Mini Kit (Agilent Technologies, Santa Clara, CA, US) and RNase-Free DNase set (Qiagen, Hilden, Germany). Sample quality and quantity were checked using the RNA 6000 Nano kit and the Agilent 2100 Bioanalyzer (Agilent Technologies), following the manufacturer’s instructions. Quantification of RNA was also confirmed spectrophotometrically, using the NanoDrop ND-1000 (Thermo Fisher Scientific, Waltham, MA, US).

### Quantitative real time–PCR (qPCR) reaction conditions and primers

First strand cDNA synthesis was run with 1 μg of total RNA and random primers, using the High-Capacity cDNA Reverse Transcription Kit (Applied Biosystems, Foster City, CA, US), and following the manufacturer’s recommendations. Reactions were prepared by adding 1 μg of total RNA, 2 μL of 10X RT Buffer, 0.8 μL of 25X dNTPs mix (100 mM), 2 μL 10X RT random primer, 1 μL of Multiscribe™ Reverse Transcriptase, and nuclease-free sterile water to 20 μL. Reaction mixtures were incubated at 25°C for 10 min, 37°C for 2 h, and 85°C for 5 s.

The qPCR experiments were run on a Stratagene Mx3000P Real-Time System (La Jolla, CA, USA) using SYBR green I with ROX as an internal loading standard. The reaction mixture was 10 μL, comprising 5 μL of 2X Maxima™ SYBR Green qPCR Master Mix (Fermentas International, Inc, Burlington, ON, Canada), 0.5 μL of cDNA and 100 nM primers (Integrated DNA Technologies, Coralville, IA, US). Controls included non-RT controls (using total RNA without reverse transcription to monitor for genomic DNA contamination) and non-template controls (water template). Specifically, PCR conditions were the following: *P. lunatus* Actin1 (*PlACT1*), Farnesyl Pyrophosphate Synthase (*FPS*), Lipoxygenase (*LOX*) [41], *P. lunatus* Ocimene Synthase (*PlOS*) [10], *P. lunatus* Terpene Synthase 2 (*PlTPS2*) [42]: 10 min at 95°C, 45 cycles of 15 s at 95°C, 30 s at 55°C, and 30 s at 72°C, 1 min at 95°C, 30 s at 55°C, 30 s at 95°C. Fluorescence was read following each annealing and extension phase. All runs were followed by a melting curve analysis from 55°C to 95°C. The linear range of template concentration to threshold cycle value (Ct value) was determined by preparing a dilution series, using cDNA from three independent RNA extractions analyzed in three technical replicates. Primer efficiencies for all primer pairs were calculated using the standard curve method [71]. Two different reference genes (Actin1 (*PlACT1*) and the *18S* ribosomal RNA) were used to normalize the results of the qPCR. The best of the two genes was selected using the Normfinder software [72]; the most stable gene was *P1ACT1*. Primers used for qPCR were as described elsewhere [3,41,42] and are reported in Additional file 5.

All amplification plots were analyzed with the Mx3000P™ software to obtain Ct values. Relative RNA levels were calibrated and normalized with the level of *PlACT1* mRNA.

### Statistical analyses

Analysis of variance (ANOVA) and the Tukey test were used to assess difference between treatments and control. For all other experiments, at least five samples per treatment group entered the statistical data analysis. PV chemical data are expressed as mean values ± standard error of the mean (SEM).

Principal Component Analysis (PCA) was used in three different steps, each targeting different discrimination (control-damage, different damage, different leaf areas). A log-transformation was used as GC-MS data pre-treatment [40]. Each PCA step was followed by a significance test for the discriminant compounds. To compare the different leaf areas, the Kruskal-Wallis test was applied to both chemical and gene expression data. Bonferroni adjustment (p/*k*; *k* = number of comparisons) was applied to protect against Type I Error [73,74].

All statistical data analyses were done using SPSS software for Windows.

## Additional files

Additional file 1:
**PV DC-STE-GC-MS profile.** The typical GC-MS profile of PVs captured by DC-STE on *Phaseolus lunatus* leaves wounded by *Spodoptera littoralis* obtained after thermal desorption of DC-STE. A compound table is also provided. Additional file 2:
**PCA analysis of time-course experiments on different damage dataset.** Score and loading plots presented in Figure 4 are here displayed taking into account sampling time of every sample (instead considering the distance from the wounded area). Additional file 3:
**Parameters for the volatiles’ quantitation.** A table reports HIPV quantitation parameters obtained by Gas Chromatography – Selected Ion Monitoring – Mass Spectrometry (GC-SIM-MS). Additional file 4:
**Camphor effects on** ***Phaseolus lunatus*** **.** A table and a barchart report a comparison between PVs in plants analyzed with and without camphor preloading on tapes. Additional file 5:
**Gene-specific primers used for quantitative real-time PCR.** A table collecting GenBank accession number and sequences of every primer used.

## Abbreviations

DAMP: Damage associated molecular pattern
DC-STE: Direct contact-sorptive tape extraction
DMNT: 4,8-dimethyl-1,3,7-nonatriene
GC: Gas chromatography
GLV: Green leaf volatile
HIPV: Herbivory-induced plant volatile
HW: Herbivore wounding
MD: Mechanical damage
MDOS: Mechanical damage with oral secretions
MS: Mass spectrometry
OS: Oral secretions
PDMS: Polydimethylsiloxane
PV: Plant volatile
TMTT: 4,8,12-trimethyl-1,3,7,11-tridecatetraene
