# Supplementary material for: Direct Contact – Sorptive Tape Extraction coupled with Gas Chromatography – Mass Spectrometry to reveal volatile topographical dynamics of lima bean (Phaseolus lunatus L.) upon herbivory by Spodoptera littoralis Boisd
Source: BMC Plant Biol. 2015 Apr 12;15:102. doi: 10.1186/s12870-015-0487-4 (PMC4415311; doi:10.1186/s12870-015-0487-4)
Supplement: Additional file 1: — PV DC-STE-GC-MS profile. The typical GC-MS profile of PVs captured by DC-STE on Phaseolus lunatus leaves wounded by Spodoptera littoralis obtained after thermal desorption of DC-STE. A compound table is also provided. [file 12870_2015_487_MOESM1_ESM.docx]

**Additional file 1: VOC DC-STE-GC-MS profile.**

The typical GC-MS profile of VOCs emitted by *Phaseolus lunatus* leaves wounded by *Spodoptera littoralis* obtained after thermal desorption of DC-STE. ISTD, Internal standard (camphor).

| **n** | **R.T. (min)** | **Compound** | **CAS** | **IUPAC** |
| --- | --- | --- | --- | --- |
| 1 | 5.617 | n-hexanal | 66-25-1 |  |
| 2 | 6.734 | (*E*)-2-hexenal | 6728-26-3 |  |
| 3 | 6.806 | (*Z*)-3-hexen-1-ol | 928-96-1 |  |
| 4 | 10.495 | 1-octen-3-ol | 3391-86-4 |  |
| 5 | 10.774 | 6-methyl-5-hepten-2-one | 110-93-0 |  |
| 6 | 11.347 | octanal | 124-13-0 |  |
| 7 | 11.519 | (*Z*)-3-hexen-1-ol acetate | 3681-71-8 |  |
| 8 | 12.150 | *p*-cymene | 99-87-6 | Methyl(1-methylethyl)-benzene |
| 9 | 12.300 | limonene | 138-86-3 | 1-Methyl-4-(1-methylethenyl)-cyclohexene |
| 10 | 12.372 | 2-ethyl-1-hexanol | 104-76-7 |  |
| 11 | 13.131 | (*E*)-β-ocimene | 13877-91-3 | 3,7-Dimethyl-1,3,6-octatriene |
| 12 | 14.084 | 1-octanol | 111-87-5 |  |
| 13 | 15.294 | linalool | 78-70-6 | 3,7-Dimethyl-1,6-octadien-3-ol |
| 14 | 15.480 | nonanal | 124-19-6 |  |
| 15 | 16.011 | DMNT | 19945-61-0 | 4,8-Dimethyl-1,3,7-nonatriene |
| 16 | 19.112 | (*Z*)-3-hexenyl butyrate | 16491-36-4 |  |
| 17 | 19.914 | decanal | 112-31-2 |  |
| 18 | 24.040 | tridecane | 629-50-5 |  |
| 19 | 30.458 | geranylacetone | 3796-70-1 | 6,10-Dimethyl-5,9-undecadien-2-one |
| 20 | 34.842 | (*E*)-nerolidol | 142-50-7 | 3,7,11-Trimethyl-1,6,10-dodecatrien-3-ol |
| 21 | 35.444 | TMTT | 101427-55-8 | 4,8,12-Trimethyl-1,3,7,11-tridecatetraene |
| ISTD | 17.121 | camphor | 464-49-3 | 1,7,7-Trimethyl-bicyclo[2.2.1]heptan-2-one |
