# Supplementary material for: Direct Contact – Sorptive Tape Extraction coupled with Gas Chromatography – Mass Spectrometry to reveal volatile topographical dynamics of lima bean (Phaseolus lunatus L.) upon herbivory by Spodoptera littoralis Boisd
Source: BMC Plant Biol. 2015 Apr 12;15:102. doi: 10.1186/s12870-015-0487-4 (PMC4415311; doi:10.1186/s12870-015-0487-4)
Supplement: Additional file 2: — PCA analysis of time-course experiments on different damage dataset. Score and loading plots presented in Figure 4 are here displayed taking into account sampling time of every sample (instead considering the distance from the wounded area). [file 12870_2015_487_MOESM2_ESM.docx]

**Additional file 2: PCA analysis of time-course experiments on different damage dataset**.

A, HW score plot; B, HW loading plot; C, MDOS score plot; D, MDOS loading plot; E, MD score plot; F, MD loading plot. Compound legend: b, (*E*)-2-hexenal; c, (*Z*)-3-hexen-1-ol; d, 1-octen-3-ol; g, (*Z*)-3-hexenyl acetate; k, (*E*)-β-ocimene; o, DMNT; p, (*Z*)-3-hexenyl butyrate; t, (*E*)-nerolidol; u, TMTT.

**A
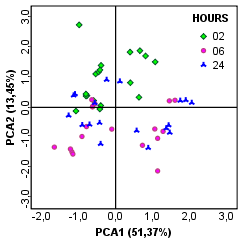
 B
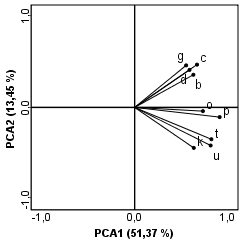
**

**C
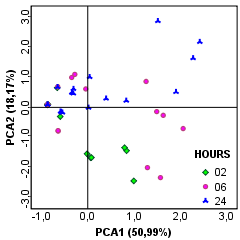
 D
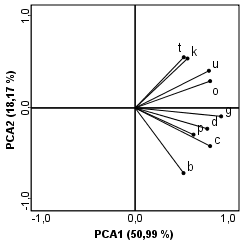
**

**E
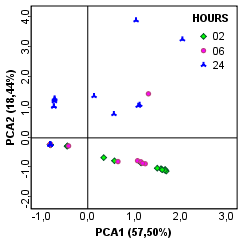
 F
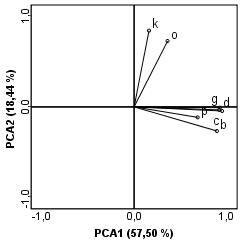
**
