# Supplementary material for: Direct Contact – Sorptive Tape Extraction coupled with Gas Chromatography – Mass Spectrometry to reveal volatile topographical dynamics of lima bean (Phaseolus lunatus L.) upon herbivory by Spodoptera littoralis Boisd
Source: BMC Plant Biol. 2015 Apr 12;15:102. doi: 10.1186/s12870-015-0487-4 (PMC4415311; doi:10.1186/s12870-015-0487-4)
Supplement: Additional file 3: — Parameters for the volatiles’ quantitation. A table reports HIPV quantitation parameters obtained by Gas Chromatography – Selected Ion Monitoring – Mass Spectrometry (GC-SIM-MS). [file 12870_2015_487_MOESM3_ESM.doc]

**Additional file 3: Parameters for the volatile quantitation.**

Parameters shown in this table were obtained by external calibration performed by GC-SIM-MS.

| **Compound** | **TGT Ion** | **Q1 Ion** | **Q2 Ion** | **calibration curve** | **R^2^** | **L.O.D. (ng/cm^2^)** | **L.O.Q. (ng/cm^2^)** |
| --- | --- | --- | --- | --- | --- | --- | --- |
| (*E*)-2-hexenal | 41 | 69 | 98 | *y* = 209.3*x* - 4707.2 | 0.9994 | 9,94 | 33,12 |
| (*Z*)-3-hexen-1-ol | 67 | 67 | 100 | *y* = 602*x* - 11627.5 | 0.9999 | 2,72 | 9,05 |
| 1-octen-3-ol | 57 | 72 | 128 | *y* = 1975.8*x* - 25617.7 | 0.9995 | 1,81 | 6,05 |
| (*Z*)-3-hexenyl acetate | 67 | 67 | 82 | *y* = 23956.2*x* - 129301 | 0.9998 | 0,16 | 0,52 |
| (*E*)-β-ocimene | 93 | 79 | 121 | *y* = 720.5*x* - 967.3 | 0.9997 | 1,05 | 3,50 |
| DMNT | 69 | 107 | 150 | *y* = 26050*x* - 197425.5 | 0.9994 | 0,07 | 0,22 |
| (*Z*)-3-hexenyl butyrate | 67 | 71 | 82 | *y* = 1037.8*x* - 1707.4 | 0.9997 | 2,77 | 9,23 |
| (*E*)-nerolidol | 69 | 93 | 204 | *y* = 370.9*x* - 13437.7 | 0.9987 | 17,01 | 56,69 |
| TMTT | 69 | 81 | 94 | *y* = 23040.3*x* - 413538.4 | 0.9976 | 0,27 | 0,90 |
