# Supplementary material for: Direct Contact – Sorptive Tape Extraction coupled with Gas Chromatography – Mass Spectrometry to reveal volatile topographical dynamics of lima bean (Phaseolus lunatus L.) upon herbivory by Spodoptera littoralis Boisd
Source: BMC Plant Biol. 2015 Apr 12;15:102. doi: 10.1186/s12870-015-0487-4 (PMC4415311; doi:10.1186/s12870-015-0487-4)

**Additional file 4: Camphor effects on *Phaseolus lunatus*.**

The table and the barchart below compare the means of integrated area of considered volatiles in plants analyzed with DC-STE tapes with or without camphor.

| **n** | **R.T. (min)** | **Compound** | **Camphor** | | **No camphor** | |
| --- | --- | --- | --- | --- | --- | --- |
|  |  |  | **Mean** | **Std. Dev.** | **Mean** | **Std. Dev.** |
| 1 | 5.617 | n-hexanal | 36276 | 39279 | 31592 | 2123 |
| 2 | 6.734 | (E)-2-hexenal | 161335 | 120517 | 172096 | 19848 |
| 5 | 10.774 | 6-methyl-5-hepten-2-one | 74900 | 10196 | 62823 | 19525 |
| 6 | 11.347 | octanal | 74195 | 1773 | 67962 | 7442 |
| 9 | 12.300 | limonene | 118445 | 18014 | 41837 | 5293 |
| 10 | 12.372 | 2-ethyl-1-hexanol | 164404 | 24740 | 181870 | 14531 |
| 12 | 14.084 | 1-octanol | 34086 | 1414 | 32849 | 8953 |
| 13 | 15.294 | linalool | 15135 | 4745 | 15697 | 2896 |
| 14 | 15.480 | nonanal | 223693 | 45934 | 252737 | 37714 |
| 17 | 19.914 | decanal | 213636 | 16190 | 202516 | 12143 |
| 18 | 24.040 | tridecane | 102603 | 17403 | 115902 | 17269 |
| 19 | 30.458 | Geranylacetone | 196904 | 28844 | 131877 | 21748 |


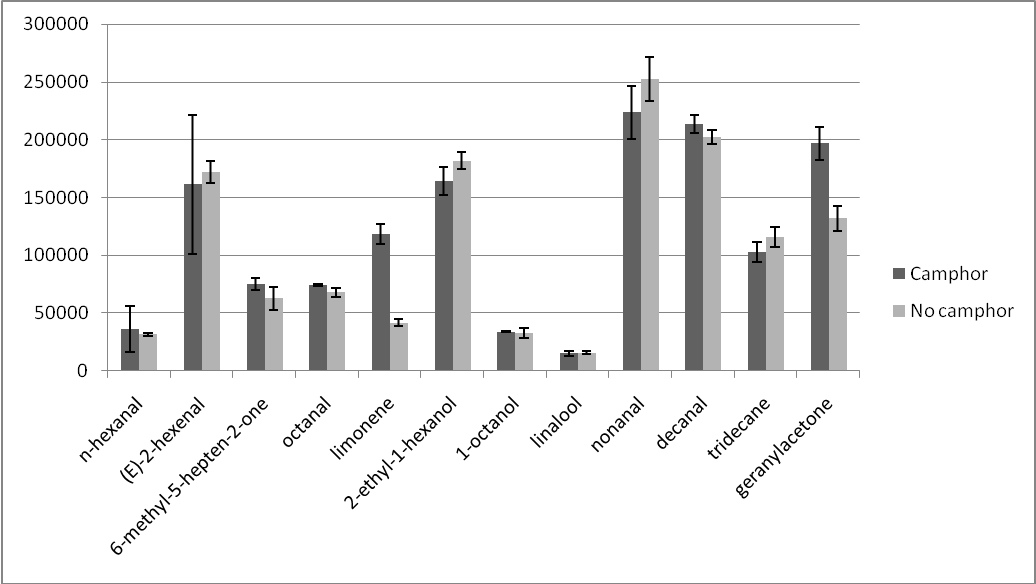

Supplement: Additional file 4: — Camphor effects on Phaseolus lunatus . A table and a barchart report a comparison between PVs in plants analyzed with and without camphor preloading on tapes. [file 12870_2015_487_MOESM4_ESM.doc]
