# Supplementary material for: Direct Contact – Sorptive Tape Extraction coupled with Gas Chromatography – Mass Spectrometry to reveal volatile topographical dynamics of lima bean (Phaseolus lunatus L.) upon herbivory by Spodoptera littoralis Boisd
Source: BMC Plant Biol. 2015 Apr 12;15:102. doi: 10.1186/s12870-015-0487-4 (PMC4415311; doi:10.1186/s12870-015-0487-4)
Supplement: Additional file 5: — Gene-specific primers used for quantitative real-time PCR. A table collecting GenBank accession number and sequences of every primer used. [file 12870_2015_487_MOESM5_ESM.doc]

**Additional file 5**: Gene-specific primers used for quantitative real-time PCR.

| **Gene** | **GenBank accession number** | **Forward primer (5’-3’)** | **Reverse primer (5’-3’)** |
| --- | --- | --- | --- |
| *PlTPS2* | KC012520 | gatgacctggaaggagacca | ggcaaactttggtgaaggaa |
| *PlOS* | EU194554 | caacaatgcatgggtctcag | tgctgcttcccctctctcta |
| *LOX* | X63521 | catggatgaccgatgaagaa | ttgctttggatagcctgctc |
| *FPS* | AB086039 | tcgttttacctcccagttgc | ttcgcaactaaccaagagca |
| *PlACT1* | DQ159907 | aggctcctcttaaccccagg | gtgggagagcataaccctc |
| *18S* | AT3G41768 | atgataactcgacggatcgc | cttggatgtggtagccgttt |
